# Neutralization by Acetyl Salicylic Acid of the Testosterone induced Impaired Maspin Synthesis Stimulated by Estriol in Neutrophils through Nitric Oxide Synthesis

**Published:** 2015-12

**Authors:** Emili Manna, Sarbashri Bank, Smarajit Maiti, Pradipta Jana, Asru K. Sinha

**Affiliations:** 1 Sinha Institute of Medical Science & Technology, Garia, Calcutta-700084, India;; 2 Department of Biochemistry, Cell and Molecular Therapeutic Lab, OIST, Vidyasagar University, Midnapur, India

**Keywords:** cross-talk, flutamide, maspin, Nitric oxide, steroid receptors

## Abstract

**Purpose:**

Maspin, an anti breast cancer protein in the mammary cell and normal neutrophil has been reported to be synthesised by the stimulation of NO production induced by estriol. The role of testosterone was investigated in the synthesis of maspin in relation to that of estriol.

**Methods:**

Fifty normal female between the ages of 25-65 years old participated in the study. Maspin synthesis was demonstrated by *in vitro* translation of maspin mRNA, followed by the quantification of maspin by enzyme linked immune absorbent assay. NO was determined by methomoglobin method.

**Results:**

Incubation of the neutrophils in HBSS both with 30 nM estriol resulted in the synthesis of 1.8 ngm maspin with simultaneous increase of NO synthesis. In contrast incubating neutrophils with 20 nM testosterone in the presence of estriol inhibited maspin synthesis to 0.33 nM with simultaneous inhibition of NO synthesis from 1.89 nM to 0 nM at the same time. Addition of 0.2 μM flutamide, a testosterone receptor blocker to the incubation mixture restored the synthesis of maspin by 60.64 %. Incubation of 25 μM aspirin that stimulated NO synthesis restored the inhibition of maspin synthesis by testosterone by 79.1%. *I*-NAME, an inhibitor of nitric oxide synthase, abolished both maspin and NO synthesis. Scatchard plot of estriol binding in the presence of testosterone demonstrated that the male sex hormone inhibited the female sex hormone binding to its receptor by “cross talk” between the receptors. It was found that while 1.02 × 10^3^ molecules of estriol bind each neutrophil at equilibrium, in the presence of testosterone (20 nM) in the binding mixture decreases the binding of estriol to 0.5 × 10^3^ with little change in the dissociation constant compared to controls.

**Conclution:**

Estriol was found to stimulate maspin synthesis through the stimulation of NO, testosterone inhibited maspin synthesis through the inhibition of NO synthesis.

## INTRODUCTION

MASPIN (Mammary Serine Protease Inhibitor), a member of the serpin super family has been reported to play an important role in the prevention and control of breast cancer (1). This mammary epithelial derivative anticancer protein has been reported to have anti metastatic and anti-invasive properties (2) as well as has been reported to increase apoptosis (programmed cell death) in breast cancer cells (3). This protein has been demonstrated to be a product of a tumor suppressive gene. When exogenously added, a recombinant maspin has been reported to alter the invasive property of the breast cancer cells (4). Interestingly, the nuclear location of the tumor suppressor gene activity of maspin has been reported to inhibit breast cancer cells without affecting the proliferation of the normal epithelial cells in the breast (5). It has been suggested that endogenously synthesized maspin could be a therapeutic target agent even for triple negative estrogen and progesterone receptors statuses in breast cancer (6), where the prognosis of the condition has been reported to be worst compared to that of the double negative estrogen receptors statuses (7). The recombinant maspin, used in liposome carrier, had been reported to inhibit lung metastasis (8).

In the above context that maspin can reduce the development of the breast cancer; the role of high plasma testosterone has been reported to impute the occurrence of breast cancer (9). The presence of free testosterone in the circulation has been positively associated with increased breast cancer risk (9). In contrast, we have reported before that estriol (an estrogen) could have beneficial effect on the breast cancer due to maspin synthesis through the direct activation of nitric oxide synthase by the estrogen (10). The role of systemic interplay between the testosterone and estriol in the synthesis of maspin could have important consequences in the development on breast cancer.

We herein report that a “cross talk” (11) between the receptors of estriol and testosterone could have important input in the estriol induced stimulation of maspin synthesis in the neutrophils. The “cross-talk” between the receptors of two different hormones (ligands) implies that the binding of one of the two different ligands to its own receptors on the cell surface may sometime increase or decrease the receptors of density of other ligands on the same cells surface. It was originally thought that the increase or decrease of the receptor density on the cell surface due to the binding of different ligand to its own receptors was related to the synthesis or inhibition of the receptor protein synthesis in the cell. It was latter on however demonstrated that the “cross-talk” between the receptors could be independent of the protein synthesis but could be related to the exposure of the “spare” receptors from the lipid membrane bilayers to the cell surface (12). We further demonstrated that acetyl salicylic acid (aspirin) was capable of neutralizing the inhibitory effect of testosterone in the synthesis of maspin due to the cross-talk between the steroid receptors in the neutrophils.

## METHODS

### Ethical Clearance

The study involved in the participation of the normal female volunteers for the donation of blood sample (20 ml).The permission was obtained from Internal Review Board. All blood donors were asked to sign inform consent form before they were requested to donate blood sample. This study also raised polyclonal antibody in white Newzealand rabbits. Appropriate permission was also obtained from the IRB for the animal study.

### Selection of volunteers

Only female volunteers between the age of 25 to 55 years old (n=50) participated in the study. These volunteers at presentation had no systemic hypertension or suffer from diabetes mellitus. These participants has not been hospitalised for any condition at least for 6 months before they were allowed to participate. None of the volunteers had received any aspirin or any preparation containing aspirin at least for 14 weeks before they participated in the study. None of the volunteers had ever received any contraceptive medication. These participants were from all walks of life including school teachers, office workers, students and house wives.

### Collection of blood sample

Peripheral blood samples were collected by venipuncture using 19 gauge siliconized needles, and the blood samples were collected in plastic tube and anticoagulated by using sodium citrate, mixed by gentle inversion of the plastic tube.

### Chemicals and materials

Aspirin (acetyl salicylic acid) was purchased from medica zudas health care; *I*-NAME (N^G^-nitro-L-arginine methyl ester), Flutamide (2-methyl-N-[4-nitro-3-(trifluoromethyl) phenyl] propanamide), testosterone and estriol were from Sigma Aldrich. Maspin was a gift from Dr. Sally Twining of the Dept. of Biochemistry. Polyclonal maspin antibody was developed in rabbit (13). Testosterone and estriol were purchased from Sigma. Glass microfibre membrane was from Whatman International ltd. Enzyme linked immunosorbent assay (ELISA) maxsorb plate was from Nunc Roskilde. All the chemicals were of analytical grade.

### Preparation of aspirin solution

Aspirin was prepared by dissolving the compound in 0.9% NaCl solution and ph 7.4 was adjusted by the addition of 0.1mM NaHCO_3_ immediately before use and discarded after use.

### Preparation of Flutamide solution

Flutamide (2-methyl-N-[4-nitro-3-(trifluoromethyl) phenyl] propanamide) was prepared by dissolving the compound in 0.9% NaCl solution.

### Preparation of Estriol solution

As it is steroid in nature, estriol solution was prepared by dissolving the compound at first in 100 μl of isopropyl alcohol and then the total solution was dissolved in the 0.9% NaCl solution, and pH was adjusted to 7.4. After completion of preparation the solution was used immediately and after use it was discarded.

### Preparation of Testosterone solution

Testosterone solution was prepared by dissolving it at first in 100 μl of isopropyl alcohol, and then 0.9% NaCl solution, maintaining the pH 7.4 and used immediately. The solution was discarded after use.

### Preparation of neutrophil suspension and the incubation of the isolated neutrophils with estriol and testosterone

The neutrophil cell suspension was carried out by isolating nutrophils from the citrated blood samples as described previously (14). The cells count was studied by using optical microscopy. The isolated neutrophils (10^6^/ml) suspended in Hank’s balanced salt solution (HBSS), pH 7.4 were incubated with different concentrations of estriol for 30 minutes or different concentrations of testosterone for 2 h at 37°C under sterile conditions.

### Ribosomal particle preparation from bael leaves

Bael leaves (*Aegle marmelos*) were collected and washed thoroughly by normal deionised water then were rinsed it by distilled water. The properly cleaned leaves were homogenised and leaf extract was filtered, separated and then centrifuged at 6000 rpm at 10 minutes to remove the debris. Supernatant was collected and stored it at 0°C as described (15).

### *In vitro* translation of maspin–mRNA

The synthesis of maspin was not quantitated by a direct ELISA to exclude the preformal maspin (basal) in the samples by *in vitro* translation of the corresponding mRNA followed by quantitation of the newly synthesised maspin by ELISA.

Nuclic acids were extracted from the isolated neutrophils containing maspin mRNA was carried by using Trizol (16). The extracted nucleic acids were incubated with the ribosomal particle, amino acids mixture of all 20 amino acids (0.1 μmol each /ml) and 2 mM ATP for 6 hours as described (17). The incubation mixture was used for the determination of maspin by ELISA as described below.

### ELISA for maspin

Maspin level was determined by ELISA from the normal volunteers by using a polyclonal antibody developed against rh-maspin according to the method described previously (18).

### Assay of NO

Assay of nitric oxide was determined by the methemoglobin method by using Beckmen spectro photometer at 575-600 nm as described previously (19). The validity of the assay was confirmed independently by using a chemiluminescence method (20).

### Scatchard plot analysis of the equilibrium binding of estriol to normal neutrophils

Neutrophils were isolated by dextran (14) and preequilibrated with 0.9% NaCl (14). Isolated neutrophils were incubated with different concentrations of estriol for 120 min at 37°C. After incubation, 300 μl of reaction mixture was poured into micro glass fiber membrane (GF/C) by using Millipore filtration unit which was described before (21). GF/C membrane can help in the adherence of neutrophils and free estrogen can pass through the filtrate and the membrane was washed by 0.9% NaCl solution. Bound estriol was eluted by 1:1 CHCl_3_ and CH_3_OH mixture. The eluted material then was air dried at 37°C and amount present in elution was determined by ELISA as described before (18).

The binding of estriol to neutrophils was analysed by Scatchard plot and the dissociation constant (K_d_) and number of binding sites of estriol was calculated. Neutrophil numbers were quantitated by optical microscopy.

### Statistical analyses

The results shown here are mean ± S.D. Significance of the results was determined by two tailed pair ‘t’ test (*p*<0.05). Correlation of coefficient was analysed by pearson ‘r’ value (+1 to -1) using by Graph pad Prism software. Dissociation constant (K_d_) and B_max_ from scatchard plot analysis were demonstrated by Microsoft office excel.

## RESULTS

### Effect of Estriol in the synthesis of maspin in the neutrophils through NO synthesis

**Figure 1:**
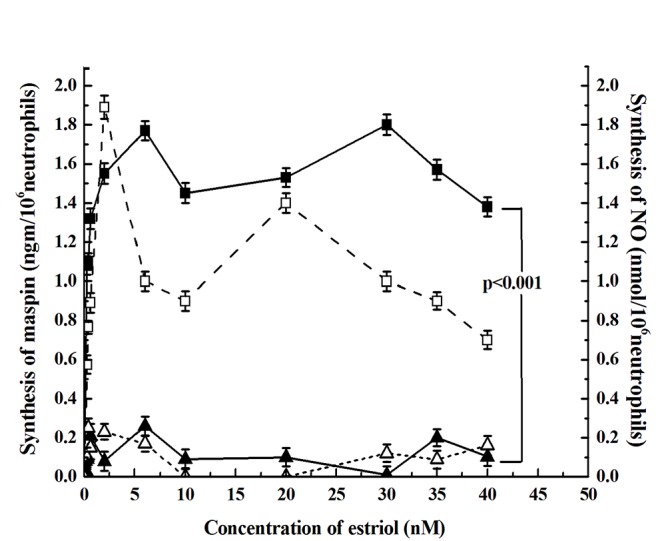
**The effect of different concentration of estriol of the neutrophils and the synthesis of NO.** Neutrophil suspension 10^6^ cells/ml in Hank’s balanced salt solution, pH 7.4 was incubated with different concentration of estriol for 30 min at 37°C as shown. After incubation the synthesis of maspin was determined by *in vitro* translation of maspin mRNA and quantitated by ELISA as described in the materials and method. While solid squares with solid line (—■—) represent the synthesis of maspin, the hollow square in the broken line (--□--) show the synthesis of NO. The hollow triangle on the broken line (--Δ--) show the synthesis of estriol induced NO synthesis in the presence of 0.1nM NAME in the incubation mixture. The solid triangle on the continuous line (—▴—) represents the maspin synthesis in the presence of 0.1nM NAME in the incubation mixture. Results shown are mean ± standard deviation 10 different experiments by using 10 different blood samples from 10 different volunteers each in triplicate.

Incubation of neutrophils with different concentrations of estriol resulted in the synthesis of maspin as determined by in vitro translation of maspin m-RNA (Fig. 1). It was found that the maximum amount of maspin was achieved at 30 nM estriol *in vitro*. The dose response curve of the estriol induced synthesis of maspin resulted in a biphasic stimulation of maspin synthesis, in that at 5 nM estriol , maspin synthesis was found significantly to be increased when compared to the control (*p*<0.001, two tailed paired t test). However farther increase of the estriol in the incubation mixture decreased production of the anti-breast cancer protein. However further increase of the estriol at 30 nM resulted in the maximal synthesis of maspin. But further increase of estriol concentration in the reaction mixture resulted in the decrease production of the protein.

### The effect of testosterone on the estriol induced maspin synthesis neutrophil

**Figure 2:**
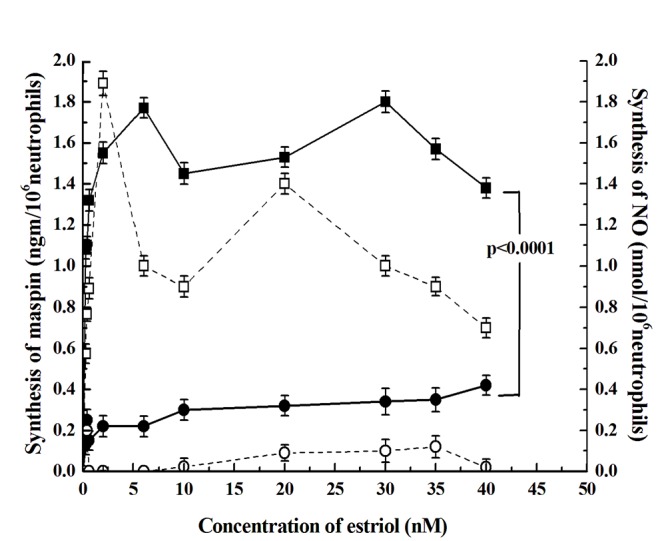
**Effect of testosterone on the estriol induced maspin and NO synthesis in neutrophils.** Neutrophils suspension was prepared as described in the method and materials. The suspension was incubated with 20nM testosterone for 2h at 37ºC. The neutrophils suspension prepared with testosterone was re-incubated with different concentration of estriol as shown for 30 minute at 37ºC. After incubation both maspin and NO synthesis were determined as described in the Materials and Method. While the solid squares with solid line (—■—) shows maspin synthesis in the absence of testosterone, the hollow squares (--□--) on the broken line represent the synthesis of NO. The hollow circles on the broken line (--o--) represents the synthesis of NO and the solid circles with solid line (—●—) show the synthesis of maspin in the presence of 20nM testosterone. The results presented are mean ± S.D. of 10 different experiments each in triplicate by using the blood sample from 10 different volunteers.

As described above elevated testosterone level caused increased breast cancer risk (11), the effect of pre-incubation of neutrophils with testosterone on the estriol induced maspin synthesis was determined. It was found that the preincubation of neutrophils with 20nM testosterone for 120 minutes at 37°C resulted in the inhibition of estriol induced stimulation of maspin synthesis at all concentrations of estriol used (from 10 nM to 40 nM) (Fig. 2) by as much as >90% (*p<0.0001*, two tailed paired t test).

### Role of testosterone receptors on the inhibition of the estriol induced maspin synthesises in neutrophils by male androgen

**Figure 3:**
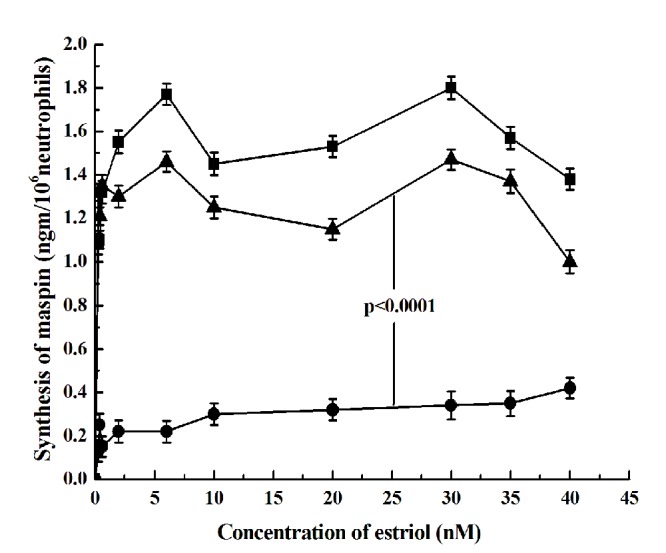
**The effect of testosterone on the synthesis of the estriol induced maspin synthesis and the effect of flutamide (testosterone receptor blocker) on the inhibitory effect of testosterone.** Neutrophil suspension in HBBS buffer was incubated with 20nM testosterone and different concentration of estriol as shown was incubated at 37°C as described under Fig. 2. After incubation the synthesis of maspin was determined. In a separated but parallel experiment the incubation mixture was treated with 0.2 µM Flutamide, a well known testosterone receptor blocker (26) and after incubation maspin synthesis was determined. The solid square solid line (—■—) represents maspin synthesis by estriol. The solid circle (—●—) represents synthesis of maspin by estriol in the presence of 20 nM testosterone. Solid triangle with solid line (—▴—) represents the synthesis of maspin in the neutrophil suspension treated with 0.2 µM Flutamide. The results shown are mean ± S.D. of 10 different experiments, each in triplicate using blood samples from 10 different female volunteers.

To determine whether the testosterone induced inhibition of the estriol stimulated maspin synthesis was actually related to the activation of the testosterone receptors by the male sex hormone in the neutrophils, flutamide (0.2 μM) was added to the incubation mixture. The addition of flutamide, a well known testosterone receptor blocker (22) was found to reverse the inhibitory effect of testosterone on the estriol stimulated maspin synthesis (Fig. 3) (*p*< 0.0001, two tailed paired t test).

### The role of nitric oxide on the stimulatory effect of estriol and in the inhibitory effect of testosterone in maspin synthesis in neutrophils

**Figure 4:**
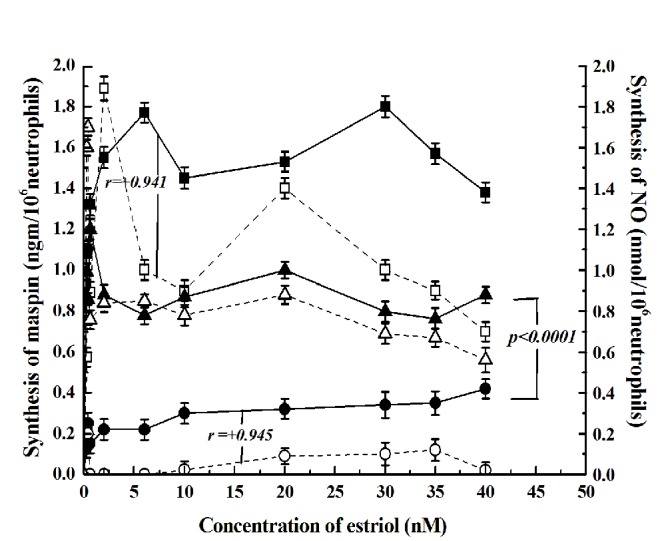
**The effect of testosterone on the estriol induced maspin and NO synthesis and the effect of addition of NO solution in 0.9% NaCl on the inhibitory effect of testosterone to the incubation mixture on the testosterone.** Neutrophil suspension was prepared with 20 nM testosterone followed by incubation of the suspension with different concentration of estriol as shown. After incubation the synthesis of both NO and maspin was determined. In a separate experiment NO solution in 0.9% NaCl was added to the reaction mixture and the synthesis of estriol induced maspin in the presence of testosterone was carried out. In the control experiment equal volume of 0.9% NaCl was added to the reaction mixture. The solid square with solid line (—■—) represents maspin synthesis the hollow square (--□--) on the broken line show the NO synthesis in the presence estriol. The hollow circle on the broken line (--o--) represents the synthesis of NO and the solid circles (—●—) show the synthesis of maspin in the presence of 20 nM testosterone. The hollow triangle (--Δ--) shows the synthesis of NO and the solid triangle with solid line (—▴—) represents the synthesis of maspin in the presence of 0.8 nmol NO in 0.9% NaCl. The results shown are mean ± S.D. of 10 different experiments, each in triplicate using blood samples from 10 different female volunteers.

We have reported before that increase of NO synthesis resulted in the synthesis of maspin in neutrophil (23). To determine the effect of estriol on the NO synthesis as related to the synthesis of maspin (as described under Fig. 1), when different concentration of estriol was added to the neutrophil suspension incubated for 30 minutes (optimum time determined in separate experiment) the synthesis of NO was found to follow parallel increase of maspin synthesis (Fig. 4). In contrast addition of 20 nM testosterone in the incubation mixture not only resulted in the inhibition of maspin synthesis but also inhibition of the NO synthesis (Coefficient of correlation *r =+0.945, r =+0.941*). On the other hand addition of 0.1 mM *I*-NAME, (inhibitor of NO synthase) completely inhibited the estriol induced maspin synthesis. However addition of 1 nM NO in 0.9% NaCl to the reaction mixture was able to reverse the inhibitory effect of 20 nM testosterone on the estriol induced maspin synthesis (from 0.3 ngm maspin/10^6^ neutrophil to 0.8 ngm maspin/10^6^ cells) even in the presence of testosterone (*p*<0.0001).

### Effect of Acetyl Salicylic Acid (Aspirin) on the nullification of the testosterone induced inhibition of maspin synthesis in neutrophils

**Figure 5:**
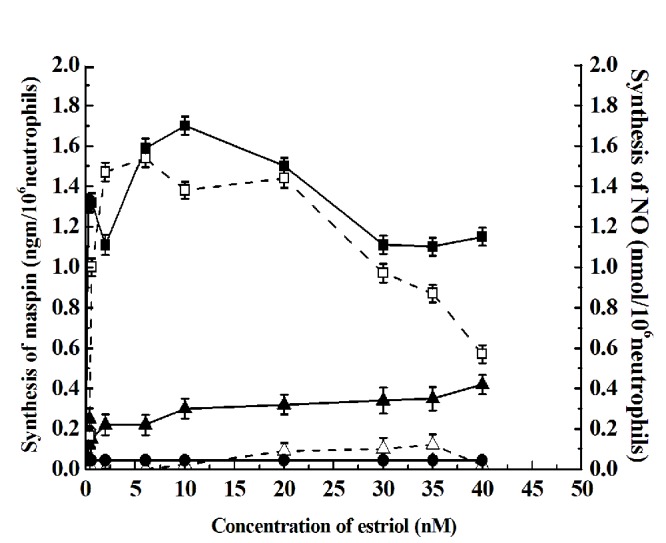
**Effect of acetyl salicylic acid on the testosterone induced inhibitory effect on the estriol induced maspin synthesis due to NO synthesis in neutrophils.** Normal neutrophil suspension was treated with 20 nM testosterone and subsequently treated with different concentration of estriol as described in Materials and Method. In separate experiments the incubation mixture was treated with 25 μM acetyl salicylic acid. After incubation both the synthesis of maspin and NO was determined. The solid triangle with solid line (—▴—) with solid line represents synthesis of maspin and the hollow triangle (--Δ--) with broken line represents the synthesis NO in presence of 20 nM testosterone and different concentration of estriol. The solid square (—■—) with solid line represents synthesis of maspin and the hollow square (--□--) with broken line represents the synthesis NO induced by 25 μM aspirin also in presence of 20 nM testosterone and different concentration of estriol. The solid circle (—●—) with solid line represents the inhibition of maspin and NO synthesis induced by 25 µM aspirin in presence of 20 nM testosterone and different concentrations of estriol by 0.1 mM NAME.

As described under the Fig. 4, the addition of NO solution in 0.9% NaCl was capable of reversing the inhibitory effect of testosterone on the maspin synthesis in the neutrophils. We have reported before the presence of aspirin in different cell suspension *in vitro* or *in vivo* was capable of stimulating NO synthesis which was independent of the well known inhibitory effect of aspirin on the activation of clyclooxygenase. Experiments were carried out to find out the effect of addition of aspirin, through the NO synthesis, on the estriol induced maspin synthesis that was found to be inhibited by testosterone. It was found that the inhibitory effect of 20 nM testosterone that reduced the 10nM estriol induced maspin synthesis by 88% was completely nullified in the presence of 25 µM aspirin that was accompanied by simultaneous increase of NO synthesis (Fig. 5). Addition of 0.1 mM *I*-NAME, an inhibitor of nitric oxide synthase (24) to the reaction mixture completely abolished the synthesis of both NO and maspin induced by aspirin in the neutrophils.

### “Cross – talk” between the receptor of estriol and testosterone in the binding of estriol receptor in neutrophil

As described above, presence testosterone inhibited the estriol induced maspin synthesis through the inhibition of NO synthesis in neutrophils.

**Figure 6:**
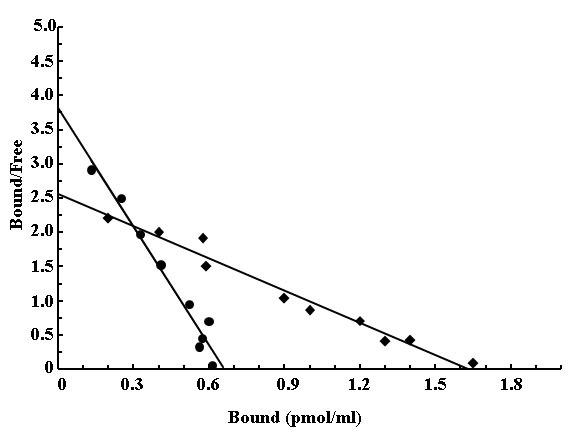
**Scatchard plots of the equilibrium binding of estriol to neutrophils in the presence and absence of testosterone.** Normal neutrophil suspension in HBSS buffer was prepared as described in the Material and methods. Equilibrium binding of different concentration of estriol on netrophil was analyzed after 30 min incubation and in presence of male sex hormone with estriol for 120 min, estriol binding was determined and the amount of the free estriol in the incubation mixture was also determined by using glass micro fibre filtrate (cationic), determined by using a milipore filtration unit as described in meterials and method. The amount of bound and free estriol was determined by ELISA.

To find out the possible regulatory control of estriol (female sex hormone) in the maspin synthesis by testosterone (male sex hormone) the “cross talk” between these different sex hormones was determined. Scatchard plot (25) analysis of the equilibrium binding of estriol to its receptor in the presence and absence of testosterone was carried out. It was found that the binding of estriol alone on neutrophil was (1.02 × 10^3^ molecules/cells), in the presence of male sex hormone testosterone the binding of estriol was (0.5 × 10^3^ molecules/cell), by more than 50% in the presence of the female sex hormone with the little or no changes of K_d_ in case of estriol alone K_d_=0.58, K_d_=1.24 for the estriol binding to the neutrophils in the presence of testosterone. (Fig. 6).

## DISCUSSION

The role of estrogens in the development of breast cancer remains both enigmatic and obscure. It has been reported that woman who has undergone overectomy for non-malignant condition and never received estrogen replacement therapy were never found to develop breast cancer. As estrogen is an ovarian hormone, these results suggested that the estrogen itself was responsible for carcinogenic effect of the hormone for development and promotion of the breast cancer compare to controls (26). On the other hand numerous demographic studies have reported that woman in their menopause are at greater risk to develop breast cancer (27). As the occurrence of menopause ceases to synthesize ovarian hormones , including estrogens, the systemic reduction of the estrogens might have been related to the development of the condition , this scenario however became complicated when it was known that woman are also at increased risk of developing breast cancer in the beginning of the menstruation (28).

These demographic studies imply that a second component beside estriol could have a critically important contribution in the development of breast cancer in woman. Indeed as presented in the text increased testosterone level in the circulation has been reported to increase breast cancer risk (9). Our results demonstrated for the first time that a biochemical quantitation of the testosterone effect in the impaired maspin synthesis induced by estriol may also occur in the pathophysiology of breastcancer.

It has been suggested that while estrogen was a probreast cancer hormone, progesterone another ovarian hormone counteracted the role of estrogens. However we have reported that at least in the case maspin synthesis, both estriol (one of the estrogens among ten) and progesterone were both capable of stimulating maspin synthesis in neutrophils (23). It should be mentioned here that the occurrence of malignancy in the mammary cells resulted in the severe impairment of maspin synthesis in neutrophils in the circulation (29).

The results as presented in the text above demonstrated that estriol induced maspin synthesis in the neurtophils was not the effect of the estrogen alone but even a well-known major male sex hormone i.e. testosterone was critically involved in the synthesis of maspin. These results might also explain, at least partly, how the free high testosterone level in the plasma might actually be involved as a risk factor for breast cancer in females (9). It was found that either in the case of the stimulation of maspin synthesis or in the case of testosterone induced inhibition of estriol induced maspin synthesis, nitric oxide (NO) played a critically important role in both cases. These results suggested that while the effect of estriol was mediated through the estrogen induced NO synthesis; the male sex hormone impaired the stimulatory effect of estriol through the inhibition of NO synthesis. Indeed, the presence of NO on 0.9% NaCl (Fig. 4) or aspirin (Fig. 5) which stimulated NO synthesis, was able to reverse the inhibitory effect of testosterone. That the effect of testosterone was actually mediated through the testosterone receptor interaction was supported by the fact that the addition of flutamide, a well known blocker of testosterone receptor (22) was able to nullify the inhibitory effect of the male sex hormone (Fig. 3). Our results also demonstrated for the first time ever that a major male sex hormone was capable of regulating the activity of a female sex hormone by controlling the binding of a female sex hormone to its estrogen receptors (cross talk) between the receptors of male and female sex hormones in the synthesis of maspin (Fig. 6).

Interestingly above mentioned male and female sex hormones were found to have opposite effect on the synthesis of NO by the catalytic activity of nitric oxide synthase (NOS). It was found that while estriol, the female sex hormone, was capable of stimulating NO through the stimulation of NOS. The male sex hormone testosterone was a potent inhibitor of NOS leading to the systemic inhibition NO synthesis.

Finally, our results also suggested that if these results could be extended to the *in vivo* conditions in humans the detrimental effect of testosterone on the estriol stimulated maspin could be overcome by consuming of 25 µM aspirin (equivalent to 22.5 mg aspirin/70 kg body weight / day) for possible prevention of breast cancer. From some studies role of aromatase in cancer has been observed. Although in our study aromatase mediated conversion of testosterone to estriol was not possible because it has been reported that aromatase is resistant in breast cancer and here its role particularly on maspin related breast cancer is out of context. It is noted that this is an *in vitro* study and here we have seen that the level of testosterone is elevated in breast cancer patients and estriol level was diminished. So, here conversion of testosterone to estriol was not happened in neutrophil cells at least in *in vitro* study.

We have reported before that the stimulation of NOS by insulin activated nitric oxide synthase (IANOS) was beneficial in breast cancer (30) and the systemic increase of aspirin induced NO synthesis that could be beneficial against the metastasis of the breast cancer, a form of cancer well known for its metastatic potential.

## CONFLICT OF INTEREST

Authors declare that they have no financial or other relationships with commercial entities whose products or services are related to the subject matter in the manuscript, or socio-political issues that can cause conflict.
